# Steviol, a natural product inhibits proliferation of the gastrointestinal cancer cells intensively

**DOI:** 10.18632/oncotarget.25233

**Published:** 2018-05-29

**Authors:** Junming Chen, Yongmei Xia, Xiaochen Sui, Qingrui Peng, Tongtong Zhang, Jian Li, Jue Zhang

**Affiliations:** ^1^ State Key Laboratory of Food Science and Technology, Jiangnan University, Wuxi, Jiangsu 214122, China; ^2^ Key Laboratory of Synthetic and Biological Colloids (Ministry of Education), School of Chemical and Materials Engineering, Jiangnan University, Wuxi, Jiangsu 214122, China; ^3^ International Joint Laboratory on Food Safety, Jiangnan University, Wuxi, Jiangsu 214122, China; ^4^ Key Laboratory of Nuclear Medicine of Ministry of Health, Jiangsu Institute of Nuclear Medicine, Wuxi, Jiangsu 214063, China

**Keywords:** steviol, steviol glycoside, cancer, gastrointestinal

## Abstract

New anticancer agents with lower toxicity have been always urged because of drug resistance associated with overused chemotherapy agents. In this study, steviol, a colonic metabolite of natural sweetener and also a component in leaves of *stevia rebaudiana bertoni*, was found to possess intensive anticancer activity on the human gastrointestinal cancer cells. Steviol inhibited six human gastrointestinal cancer cells intensively as 5-fluorouracil did at 100 μg/mL. The inhibition mechanism follows mitochondrial apoptotic pathway that was evidenced by increase of Bax/Bcl-2 ratio, activation of p21 and p53; and caspase 3-independent mechanism was also involved. These results are consistent with the miRNA expression analysis. The most regulated miRNAs in the steviol treated gastrointestinal cancer cells were miR-203a-3p (log2 =1.32) and miR-6088 (log2 =-2.54) in HCT-116, miR-1268b (log2 =19.85) and miR-23c (log2 =-2.05) in MKN-45. In view of the metabolic characteristics of steviol and its cytotoxicity on the cancer cells, steviol could be a chemotherapy agent potentially for cancer treatment.

## INTRODUCTION

Cancer has become the leading cause of mortality for Chinese population, with 4.3 million newly diagnosed cancer patients and 2.8 million deaths in 2013 [1, 2], but cancer incidence is still increasing rapidly, esspecially for gastrointestinal cancers.

Most treatments on gastrointestinal cancers execute chemotherapy either before or after surgery. The anticancer drugs, such as docetaxel, doxorubicin (DOX), 5-fluorouracil (5-FU), and cisplatin (diammine dichloroplatinum (II), CDDP), have drawbacks of non-specificity, drug resistance, and toxicity on normal cells. To overcome these limitations, either new drug discovery or combinatorial chemotherapy urges finding competitive anticancer agents.

Steviol, a rare component in leaves of *stevia rebaudiana bertoni*, is also the only colonic metabolite of steviol glycosides—a family of natural sweeteners. To date, about 40 steviol glycosides have been discovered in leaves of *stevia rebaudiana bertoni*; most of them share a similar metabolism pathway in human gastrointestinal tract [3]. The steviol glycosides are not digested until they reach colon where they are hydrolyzed to steviol. Thereafter, falling in enterohepatic circulation, some steviol is absorbed in the colon and then glucuronated in the liver; while the rest steviol is found in feces [4, 5].

So far, few reports have been found regarding the cytotoxicity of steviol on human cells. Previously, we reported that steviol presented competitive anticancer activity on U2OS cells [6]. Comparing to 5-FU (LD 50 of 115 mg/kg, oral, mouse) and DOX (LD 50 of 14 mg/kg, oral, mouse), steviol possess a much higher LD 50 of 15 g/kg BW in rats and mice (both sexes) [7] and a ADI of 4 mg/kg BW/day [8]. During the metabolism of steviol or steviol glycosides, steviol cannot be detected in the blood and its duration in human body is much longer than that of 5-FU and DOX. Therefore, if steviol presented anticancer activity on more cancer species, it may be served as a potential chemotherapy agent for cancer treatment; moreover, steviol glycosides could be found themselves used in targeting chemotherapy agent rather than as a natural sweetener. Thus, in this study, the *in vitro* anti-proliferation of steviol on the human gastrointestinal cancer cells were investigated, and the mechanism was studied with expression analysis on the relative proteins and miRNA.

## RESULTS AND DISCUSSION

### Inhibition of steviol on viability of the gastrointestinal cancer cells

**Figure 1:**
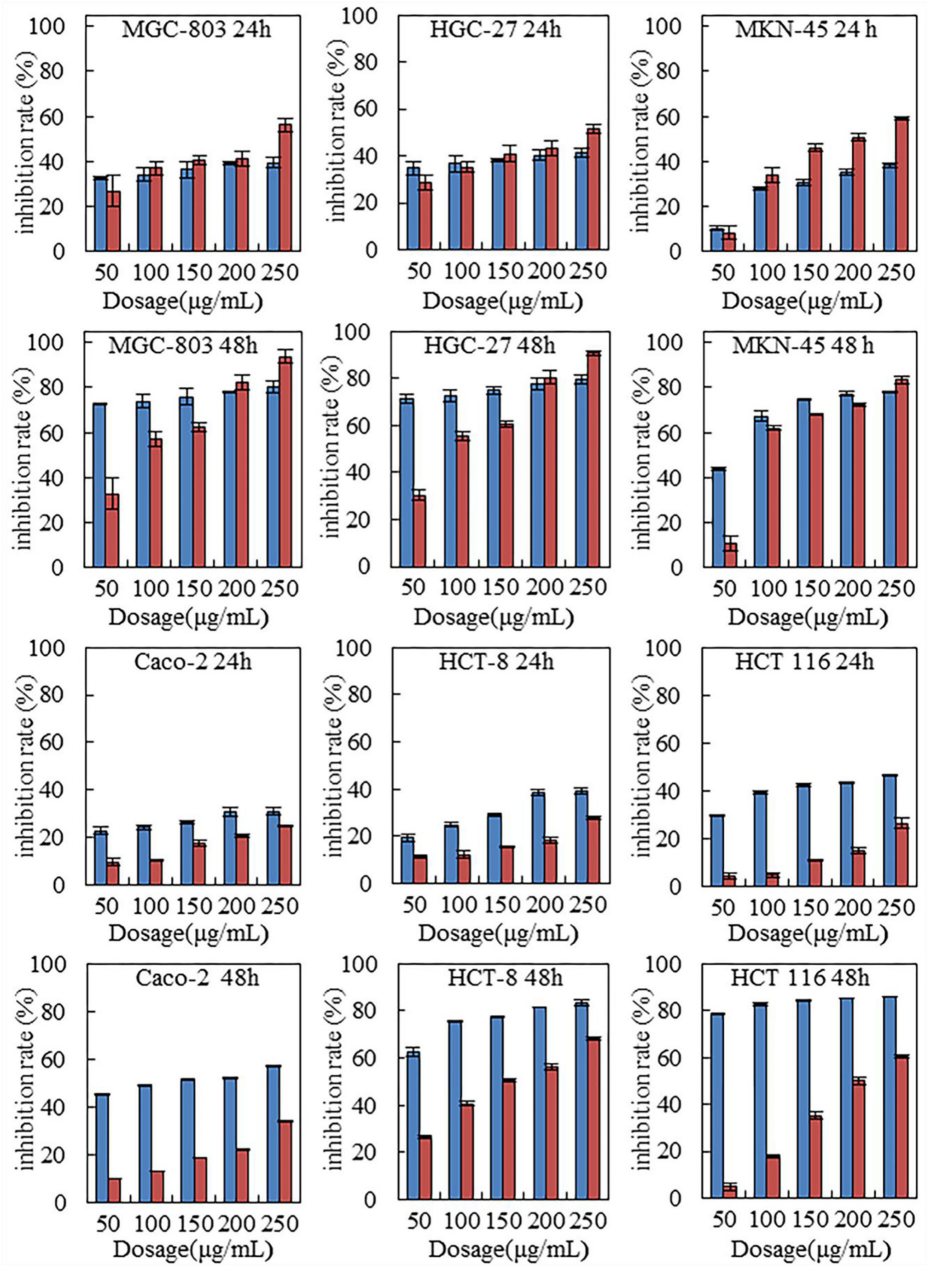
Steviol inhibited proliferation of the human gastrointestinal cancer cells

Six human gastrointestinal cell lines were tested with steviol treatment. For comparison, 5-FU was used as the positive control. Figure 1 indicates that steviol inhibited the cells viability in a time and dose-dependent manner. With same dosage (100-200 μg/mL), steviol presented a similar inhibition efficiency as 5-FU did on all assayed cancer cells; at 250 μg/mL, steviol even performed stronger inhibition. Considering that steviol possess a LD 50 value of 130 times higher than that of 5-FU, this is a remarkable performance indeed.

### Steviol causes phase arrest and apoptosis of the gastrointestinal cancer cells

**Figure 2:**
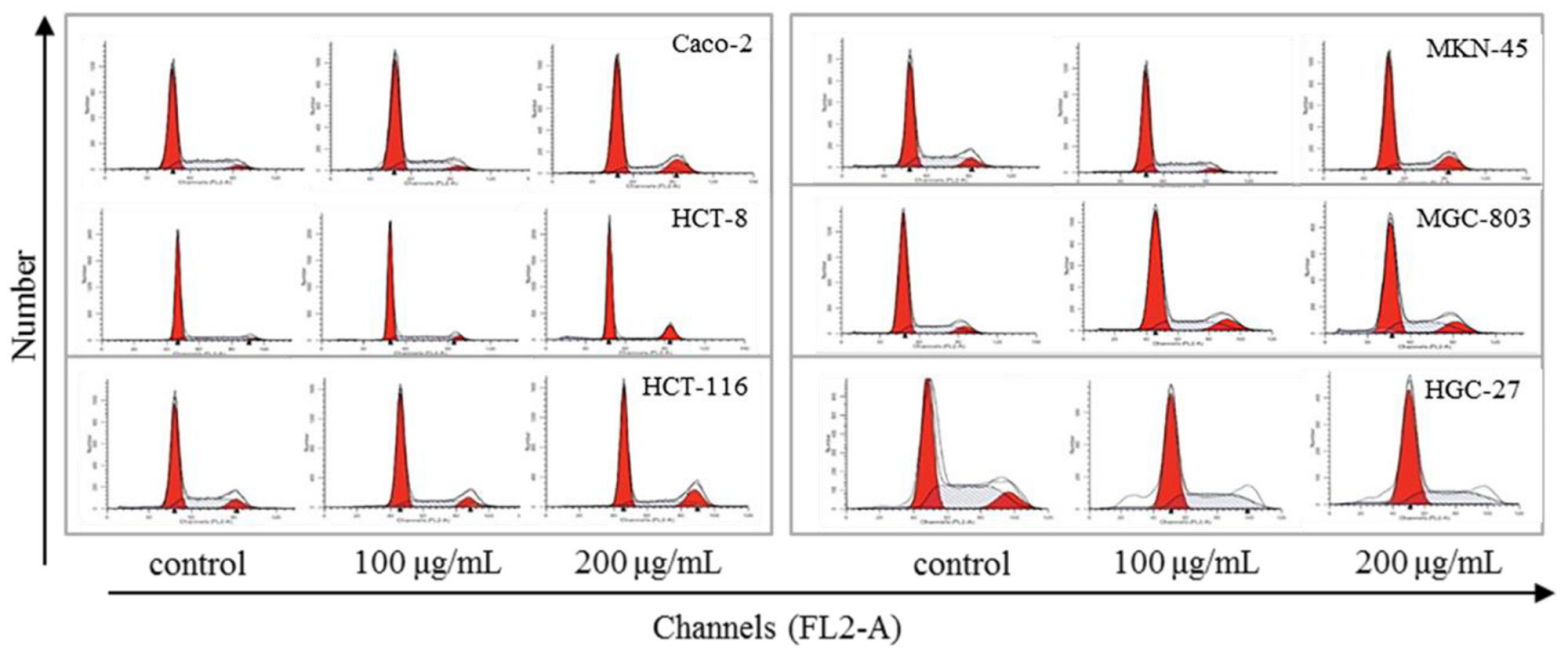
Effect of steviol on cell cycle distribution in the human gastrointestinal cancer cells

Many anticancer agents inhibit cancer cells via cell phase arrest and cell apoptosis pathway. Exposure to 1000 ng/ml of 5-FU in SW480 and COLO320DM resulted in G1-S-phase arrest and induction of apoptosis [9]. 5-FU induced apoptosis of the gastric cancer cells, and up-regulated Bax/Bcl-2 ratio in MKN-74 and MKN-45 cell lines [10, 11]. Low-dose cisplatin induced a transient G1-S phase arrest and apoptosis in HepG2 by down-regulation of p27KIP1 and Bcl-xL [12]. Similarly, as Figure 2 and Table 1 indicated, steviol also caused cell phase arrest and apoptosis. Specifically, steviol treatment caused G1 arrest on Caco-2, HCT-116, MKN-45 and HGC-27, G2 arrest on HCT-8 and MGC-803, respectively. Therefore, HCT-116, MKN-45 were selected as representative of gastric and colonic cancer cells, respectively, for subsequent experiments on cell apoptosis and miRNA analysis thereafter.

**Table 1:** Effect of steviol on cell cycle progression of the gastrointestinal cancer cells

Cell	Dosage (μg/mL)	G1 (%)	S (%)	G2 (%)
Caco-2	Control	62.55	32.69	4.77
	100	65.69	29.39	4.91
	200	70.89	21.22	8.00
HCT-8	Control	73.05	24.41	2.55
	100	71.69	23.32	4.99
	200	81.94	10.06	8.00
HCT-116	Control	57.43	34.57	8.00
	100	65.51	26.49	8.00
	200	67.39	25.15	7.46
MKN-45	Control	57.43	34.57	8.00
	100	62.55	32.69	4.77
	200	70.89	21.22	8.00
MGC-803	Control	67.66	24.40	7.95
	100	63.71	24.57	11.72
	200	59.28	27.81	12.62
HGC-27	Control	45.05	43.81	11.15
	100	52.87	40.02	7.11
	200	57.89	31.04	11.07

**Figure 3:**
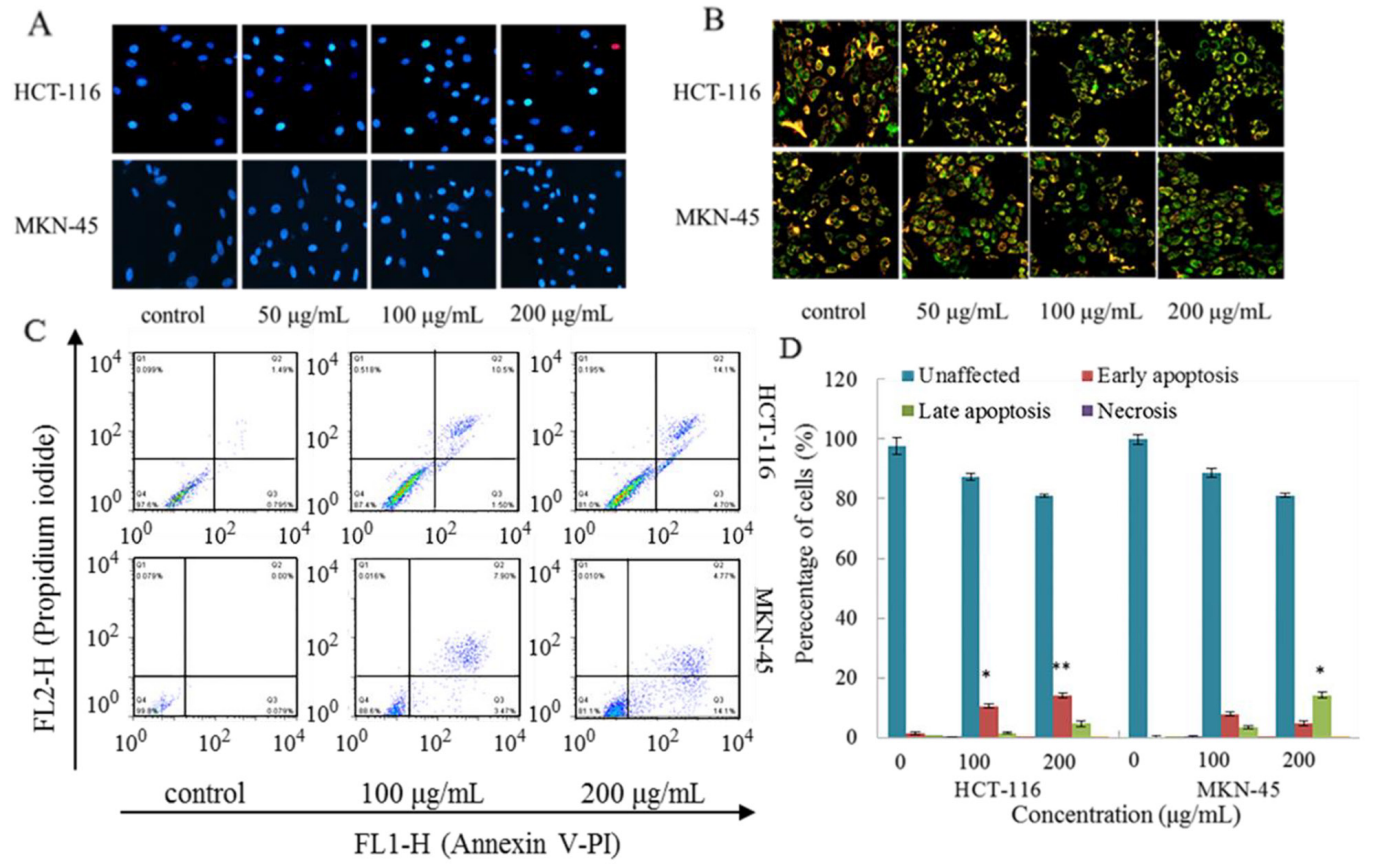
Steviol induced apoptosis of the cancer cells **(A)** Photomicrographs of Hoechst 33342. **(B)** Effect of steviol on mitochondrial membrane potential (ΔΨm) in the cancer cells. **(C, D)** the cancer cells were treated with steviol for 48 h and stained with Annexin V - PI. ^*^p<0.05, ^**^p<0.01.

The results from Hoechst staining (Figure 3A), JC-1 staining (Figure 3B), and Annexin V-FITC/PI double-labeled flow cytometry (Figure 3C, 3D) indicate a cell apoptosis in concentration dependent. These results further elucidate that steviol inhibits viability of HCT-116, MKN-45 cells through arresting the G1 phase of cell cycle, and stimulating the apoptosis progress.

Subsequently, to understand the underlining mechanism of cell cycle regulation induced by steviol, the cell cycle and apoptosis related proteins were examined in following experiment.

### Apoptotic pathway

**Figure 4:**
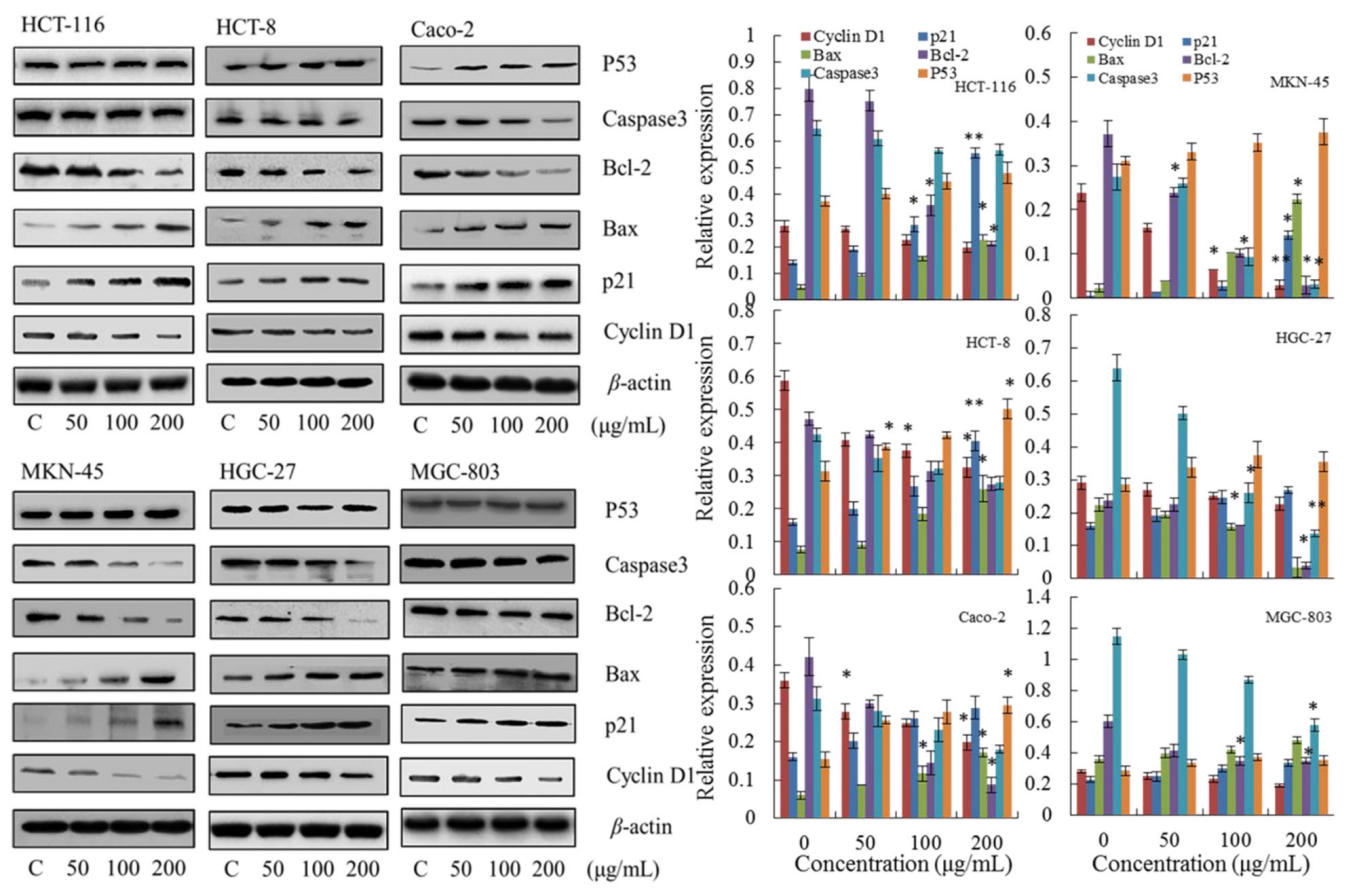
Effect of steviol on protein expression of the cell cycle and apoptosis *β*-actin was used as an internal control. ^*^p<0.05, ^**^p<0.01.

First, to investigate the mechanism of steviol-mediated phase arrest, we assessed the phase regulation related proteins including p21, p53 and Cyclin D1. As shown in Figure 4, the expression of p21 and p53 were up-regulated, whereas Cyclin D1 was down-regulated; and all of the regulations were concentration dependent.

Subsequently, the apoptosis related proteins Bcl-2, Bax and Caspase 3 in steviol treated cancer cells were detected using Western blotting (Figure 4). It reveals that the ratio of Bax/Bcl-2 was up-regulated with increasing concentration of steviol, indicating a mitochondrial apoptotic pathway [13].

Taking together, steviol has a wide-spectrum inhibitory activity on the human gastrointestinal cancer cells through leading a mitochondrial apoptotic pathway as evidenced by increase of the Bax/Bcl-2 ratio, activation of p21, p53; whereas Caspase 3-independent mechanism was involved.

### miRNA regulation on the gastrointestinal cancer cells exposed to steviol

MicroRNAs are specific for multiple cellular functions, including cell generation, differentiation, multiplication, carcinogenesis, and apoptosis. miRNAs regulation is critically involved in the development and progression of cancer [14–16]. Restoration or repression of miRNAs’ expression and activity shows high potential in managing cancer, and many studies on pre-clinical models have demonstrated the feasibility and efficacy of miRNA-based therapy [17].

To obtain comprehensive information of miRNA differential expression, human miRNA-based array layout was chosen and designed to verify the miRNA differential expression of steviol treated HCT-116 and MKN-45 cells (see also Supplementary Tables 1 and 2). As presented in Supplementary Tables 1 and 2, steviol treated cells exhibited different miRNA regulation, the most regulated as like miR-203a-3p (log2 =1.32) and miR-6088 (log2 =-2.54) in HCT-116, miR-1268b (log2 =19.85) and miR-23c (log2 =-2.05) in MKN-45. And the target gene possibly included TP53INP1 (tumor protein p53 inducible nuclear protein 1), TNFSF15 (tumor necrosis factor (ligand) superfamily, member 15) and TPD52 (tumor protein D52) for miR-203a-3p; TNFRSF11A (tumor necrosis factor receptor superfamily, member 11a, NFKB activator) for miR-6088; TUSC1 (tumor suppressor candidate 1), ZNF (zinc finger protein) and TP73 (tumor protein p73) for miR-1268b;WT1 (Wilms tumor 1), TPD52 (tumor protein D52), ST7L (suppression of tumorigenicity 7 like) and TUSC2 (tumor suppressor candidate 2) for miR-23c [18–22].

Other miRNAs regulations on the gastrointestinal cancer cells reported in literatures are summarized in Table 2 for comparison and discussion. For example, miR-146a-5P (Log2 =1.88) has a critical role in the process of AIPC prostate cancer cells apoptosis through regulation of ROCK/Caspase 3 pathway, and caspase 3 activity was stimulated by miR-146a overexpression [23]. Overexpression of miR-146b-5P (log2 =1.43) in glioma cell lines reduced cell proliferation and increased caspase-3/7 activity [24]. These results are consistent with the aforementioned protein analysis in this research.

**Table 2:** miRNAs in the gastrointestinal cancer cells presented significant differential expression

miRNA	Source	Regulation	Drug	Reference and function suggested if any
miR-663a	HCT-116 cells	upregulated	AMPs	miR-663a regulates growth of colon cancer cells, after administration of antimicrobial peptides, by targeting CXCR4-p21 pathway [31]
miR-409-3p	HCT 116, DLD-1, SW480, HT-29	upregulated	oxaliplatin	miR-409-3p is capable of enhancing the chemosensitivity of colon cancer cells by inhibiting Beclin-1-mediated autophagy [33]
miR-3142 miR-1290 miR-20a miR-4301 miR-4286 miR-3182 miR-3142 miR-1246 miR-720	Caco-2, HRT-18	upregulated downregulated	Sorafenib	[34]
miR-181c	KATO-III, MKN-45	upregulated	Iodine-125 irradiation	[35]
miR-199a-3p miR-21 miR-223 miR-342-3p miR-126 miR-146b-5p miR-1978 miR-711 miR-1280	MKN-1, MKN-45, MKN-74	upregulated downregulated	Metformin	Metformin blocked the cell cycle in G(0)-G(1) *in vitro* and *in vivo*. This blockade was accompanied by a strong decrease of G(1) cyclins [36].

In addition, some other regulations reported in literatures were also found in this experiment. For example, miR-21-5p was found to be significantly deregulated in colorectal cancer [25]; overexpression of miR-21-5p as a predictive marker for complete tumor regression to neoadjuvant chemo radiotherapy in rectal cancer patients [26]; overexpression of miR-638 inhibited the processes of tumor angiogenesis *in vitro* and *in vivo* [27]; miR-1246 was commonly upregulated in cancer cells by treatment with SAHA and DZNep and leading to apoptosis, cell cycle arrest and reduced migration of AGS and HepG2 cells [28]; miR-663a is upregulated by administration of the human cathelicidin AMP in the colon cancer cell line HCT-116, over-expression of miR-663a caused anti-proliferative effects both *in vitro* and *in vivo* [29].

Nevertheless, not all the miRNA regulations supported the inhibition, such as HCV-induced increase in miR-146a-5p expression both promotes viral infection and is relevant for pathogenesis of liver disease [30]; the low level of miR-615-5p increased the expression of RAB24 and facilitated HCC growth and metastasis *in vitro* and *in vivo* [31]; miR-31-5p was significantly upregulated for CIMP high colon carcinomas [32]. These have to be reviewed and further investigated to find the underling mechanism.

## MATERIALS AND METHODS

### Materials and chemicals

Steviol (99% of purity, HPLC) was purchased from Sigma-Aldrich Co., Ltd. (Shanghai, China). 5-Fluorouracil (5-FU), dimethyl sulfoxide (DMSO), Na_2_CO_3_, NaHCO_3_, NaCl, KCl, Na_2_HPO_4_·12H_2_O, NaH_2_PO_4_·2H_2_O, EDTA disodium, dodecyl sodium sulfate (SDS), glycine, bromoxylenol blue, ammonium persulphate, tris (hydroxymethyl) methyl amino methane, ponceau, N,N,N, N-tetramethylethylenediamine (TEMED), xylene brilliant cyanin G (BS, G250), and phenylmethylsulfonyl fluoride (PMSF) were purchased from Sinopharm Chemical Reagent Co., Ltd. (Shanghai, China). Trypsin-EDTA solution, propidium iodide (PI), triton X-100, endonuclease (RNase A), 3-(4,5-dimethylthiazol-2-yl)-2,5-diphenyltetrazolium bromide (MTT), penicillin-streptomycin solution (100X), bovine albumin (BSA), BeyoECL Plus, polyvinylidene fluoride, RIPA lysis buffer, and 5,5',6,6'-tetrachloro-1,1',3,3'-tetraethyl-imidacarbocyasix iodide (JC-1) were purchased from Beyotime Biotechnology Co., Ltd. (Shanghai, China). DMEM medium, fetal bovine serum were purchased from Gibco Life Technologies Corporation (Carlsbad, CA, USA). Primary antibodies against p21, p53, cyclin D1, Bax, Bcl-2, caspase 3, *β-*actin, and horseradish peroxidase (HRP)-conjugated secondary antibodies were purchased from Cell Signal Technologies Inc. (Beverly, MA, USA). All other reagents were of analytical grade and used as received unless otherwise stated.

### Cell culture

Cells involved in human digestion system were employed in this experiment. The human gastric cancer cell HGC-27, human colon adenocarcinoma cell Caco-2, human ileocecal adenocarcinoma epithelial cell HCT-8, human colorectal cell HCT 116, human gastric cancer cell MKN-45, and human poorly differentiated gastric cancer cell MGC-803 were purchased from Guangzhou Cellcook Biotech Co., Ltd. (Guangzhou, China). All of the cell lines were confirmed by STR. MKN-45 were cultured in RPMI 1640 medium, other cells were cultured in DMEM medium containing 10% fetal bovine serum, 1% glutamine (200 mmol/L), penicillin (100 IU/mL), and streptomycin (100 mg/L) in a humidified 5% CO_2_ atmosphere at 37 °C before use.

### MTT assay on cell proliferation

Effect of steviol on the carcinoma cell proliferation was evaluated with MTT assay [37, 38]. The cells in logarithmic growth phase were digested with 0.25% trypsin and adjusted to 5000 cells/well using DMEM complete medium, respectively. Before steviol treatment, 100 μL of the cell suspension was pipetted into each well in 96-well plates and cultured for 24 h at 37 °C in 5% CO_2_. Subsequently, cells were incubated with steviol at 37°C in 5% CO_2_ for up to 48 h. The culture medium was then removed and 100 μL of MTT reagent (0.5 mg/mL in culture medium) was added. After another 4 h of incubation, the MTT/medium was removed and 150 μL of DMSO was added to dissolve the formazan crystals. Absorbance of the solution was recorded at 570 nm to calculate the inhibition rate on cell growth. The control cell samples were prepared without steviol treatment, using same volume of culture medium to replace steviol while the other conditions remained same. Chemotherapy agent 5-fluorouracil (5-FU) was employed as the positive contrast. All measurements were performed in triplicate. The inhibition rate was calculated as following:

$$\text{Cell growth inhibition rate}\left(\%\right)=\frac{A_{570}\text{ of control}-A_{570}\text{ of sample}}{A_{570}\text{ of control}}\times 100$$

### Cell cycle analysis

The cells were plated in 6-well plate at 2 × 10^5^/well and treated with steviol. After 48 h, the cells were then harvested with trypsin, washed, resuspended in cold PBS and fixed in cold 70% ethanol for storage at -20°C overnight. Next, the cells were washed and resuspended in PBS containing 40 μg/mL PI and 0.1 mg/mL RNase, and then incubated for 30 min at room temperature. PI-stained cells were analyzed using flow cytometer and Mod Fit LT software.

### Mitochondrial membrane potential detection and Hoechst 33342 staining assay

Mitochondrial membrane potential detection was conducted with the JC-1 assay. Briefly, after steviol treatment, cells were cultured in 24-well plates and incubated with JC-1 staining solution (5 μg/mL) for 20 min at 37°C. Cells were then rinsed twice with JC-1 staining buffer. Cells on chamber slides were scanned with fluorescence microscope.

Hoechst 33342 staining assay: The cells were seeded onto chamber slides in six-well plates at a density of 1 x 10^5^ cells per well for 24 h of incubation. The cells were cultured in DMEM supplemented with 10% of FBS and incubated at 37°C in 5% CO_2_. The medium was removed with replacement of new medium containing steviol, and cells were cultured for another 24 h at 37°C in 5% CO_2_. After removal of the medium, the cells were washed with ice-cold PBS, and fixed with formalin (4%, w/v). Cell nuclei were counterstained with Hoechst 33342 (10 mg/ml in PBS) for 10 min, and then observed and imaged by a fluorescence microscope.

### Determination of apoptotic percentage in cells

Apoptotic percentage in cells was tested using Annexin V-FITC/PI double-labeled flow cytometry: The cells were plated in six-well plate at 2 × 10^5^/well and exposed to 0, 50, 100, 200 μg/mL of steviol for 24 h. After 24 h of incubation, approximately 2 x10^6^ cells were collected, centrifuged, and resuspended in 100 μL of Annexin binding buffer, then stained with Annexin V-FITC (5 μL) and propidiumiodide (PI) (1 μL) for 15 min of incubation at room temperature. Cells were detected with a FACS Calibur flow cytometer after adding 400 μL of Annexin binding buffer.

### Western blot analysis

Steviol treated cells were digested with 0.25% trypsin and 0.2% EDTA, washed with cold PBS for three times, suspended in ice-cold RIPA lysis buffer containing 1 mM phenylmethane sulfonyl fluoride (PMSF) and incubated on ice for 30 min. The suspension was then centrifuged at 12000 g for 5 min at 4°C. The protein concentration of lysates was measured with Bradford method. Equivalent amounts of protein were separated by 10% sodium dodecyl sulfate-polyacrylamide gel electrophoresis (SDS–PAGE), and then transferred to a polyvinylidene difluoride (PVDF) membrane. Membranes were blocked for 1 h at room temperature using TBST containing 5% w/v non-fat milk, and probed with primary antibodies overnight at 4°C after washing. The membranes were incubated with secondary antibody for 1 h at room temperature. Protein bands were detected using the ChemiDoc imaging system (Bio-Rad, USA).

### miRNA profile and the statistics analysis

Microarray assay was performed using a service provider (LC Sciences). tRNA was extracted using TRK-1001(LC Sciences, USA) for size fractionating to miRNA. An oligonucleotide tag was ligated to the poly (A) tail for later fluorescent dye staining. The microarray assay (μParaflo^™^ MicroRNA microarray Assay) features, probe modification, tags and hybridization were described in the Supplementary Materials. Data were analyzed by first subtracting the background and then normalizing the signals using a LOWESS filter (Locally-weighted Regression). For the two color experiments, the ratio of the two sets of detected signals (log2 transformed, balanced) and p-values of the t-test were calculated; differentially detected signals were those with p-values less than 0.01.

### Statistical analysis

Data were expressed as mean ± SD. Multigroup comparisons of the means were carried out by one-way analysis of variance (ANOVA) test with post hoc contrasts by Student–Newman–Keuls test. All statistical analyses were performed using SPSS software. P values were two-tailed, and a value P< 0.05 was considered statistically significant.

## CONCLUSIONS

The results indicate that steviol has an intensive inhibitory activity on six human gastrointestinal cancer cells with similar efficiency as 5-FU did at 100-200 μg/mL; it performed stronger cytotoxicity on the six cells at 250 μg/mL comparing to the performance of 5-FU. The inhibition mechanism follows mitochondrial apoptotic pathway, as evidenced by increase of Bax/Bcl-2 ratio, activation of p21, p53; and caspase 3-independent mechanism is also involved. These results are consistent with the miRNA expression analysis. The steviol treated cancer cells presented remarkable miRNA regulation, such as miR-203a-3p (log2 =1.32), miR-6088 (log2 =-2.54) in HCT-116, miR-1268b (log2 =19.85), miR-23c (log2 =-2.05) in MKN-45. In view of the metabolic characteristics of steviol and its cytotoxicity on human cancer cells, steviol may become a potential chemotherapy agent for cancer treatment.

## SUPPLEMENTARY MATERIALS TABLES



## Abbreviations

5-FU: 5-fluorouracil
DOX: doxorubicin
CDDP: diammine dichloroplatinum
ADI: acceptable daily intake
LD 50: lethal dose 50%
AMP: antimicrobial peptide
PI: propidium iodide
JC-1: 5,5',6,6'-tetrachloro-1,1',3,3'-tetraethyl-imidacarbocyasix iodide
AIPC: androgen-independent prostate cancer
MTT: 3-(4,5-dimethylthiazol-2-yl)-2,5-diphenyltetrazolium bromide
BSA: bovine albumin
DMEM: dulbecco’s modified eagle medium
TP53INP1: tumor protein p53 inducible nuclear protein 1
TNFSF15: tumor necrosis factor (ligand) superfamily, member 15
TPD52: tumor protein D52
TNFRSF11A: tumor necrosis factor receptor superfamily, member 11a, NFKB activator
TUSC1: tumor suppressor candidate 1
ZNF: zinc finger protein
TP73: tumor protein p73
WT1: Wilms tumor 1
TPD52: tumor protein D52
ST7L: suppression of tumorigenicity 7 like
TUSC2: tumor suppressor candidate 2
