# Supplementary material for: Steviol, a natural product inhibits proliferation of the gastrointestinal cancer cells intensively
Source: Oncotarget. 2018 May 29;9(41):26299–308. doi: 10.18632/oncotarget.25233 (PMC5995179; doi:10.18632/oncotarget.25233)
Supplement: Supplementary file 1 [file oncotarget-09-26299-s001.pdf]

Steviol, a natural product inhibits proliferation of the gastrointestinal cancer cells intensively

SUPPLEMENTARY MATERIALS

miRNA profile experiment

The HCT-116 and MKN-45 cells in logarithmic growth phase were digested with 0.25% trypsin and adjusted to 1×10^6/mL using DMEM (MKN-45 using RPMI 1640) complete medium. Before steviol treatment, 150 µL of the cell suspension was pipetted into each well of 6-well plates and cultured for 24 h at 37°C in 5% CO_2. Cells were stimulated with 100 µg/mL steviol for 48 h. After steviol treatment, the culture medium was removed, and cells were washed with cold PBS twice and centrifuged at 1000 rpm for 5 min. After decanting the supernatant, the cells were ready for tRNA extraction. The control cell samples were prepared without steviol treatment.

The assay started from 4 to 8 µg total RNA sample were 3'-extended with a poly (A) tail using poly (A) polymerase. An oligonucleotide tag was then ligated to the poly (A) tail for later fluorescent dye staining. Hybridization was performed overnight on a µParaflo microfluidic chip using a micro-circulation pump (Atactic Technologies). On the microfluidic chip, each detection probe consisted of a chemically modified nucleotide coding segment complementary to target microRNA (from miRBase, http://www.mirbase.org/) or other RNA (control sequences) and a spacer segment of polyethylene glycol to extend the coding segment away from the substrate. The detection probes were made by in situ synthesis using PGR (photo generated reagent) chemistry. The hybridization melting temperatures were balanced by chemical modifications of the detection probes. Hybridization used 100 L 6 x SSPE buffer (0.90 M NaCl, 60 mM Na_2HPO_4, 6 mM EDTA, pH 6.8) containing 25% formamide at 34°C. After RNA hybridization, tag-conjugating Cy3 dyes were circulated through the microfluidic chip for dye staining. Fluorescence images were collected using a laser scanner (Gene Pix 4000B, Molecular Device) and digitized using Array-Pro image analysis software (Media Cybernetics).

Supplementary Table 1: miRNAs regulation on steviol treated HCT-116 cells

Reporter name	p-Value	log 2-value	Reporter name	p-Value	log 2-value
miR-203a-3p	<0.001	1.32	miR-6088	<0.001	-2.54
miR-30e-5p	<0.001	1.20	miR-4267	<0.001	-1.32
miR-21-5p	<0.001	1.12	miR-5096	<0.001	-1.26
miR-31-5p	<0.001	1.04	miR-4521	<0.001	-1.16
miR-10a-5p	<0.001	0.96	miR-3197	<0.001	-0.96
miR-151b	<0.001	0.84	miR-6727-5p	<0.001	-0.85
miR-3607-5p	<0.001	0.83	miR-3665	4.53E-03	-0.85
miR-151a-5p	<0.001	0.75	miR-4734	<0.001	-0.83
miR-183-5p	<0.001	0.73	miR-7641	<0.001	-0.70
miR-29a-3p	<0.001	0.62	miR-1260b	<0.001	-0.68
miR-425-5p	<0.001	0.59	miR-125a-5p	<0.001	-0.69
miR-200c-3p	<0.001	052	miR-1973	<0.001	-0.56
miR-103a-3p	<0.001	0.52	miR-6087	<0.001	-0.52
miR-24-3p	<0.001	0.49	miR-6089	<0.001	-0.51
miR-30b-5p	<0.001	0.49	miR-7977	<0.001	-0.47
miR-200b-3p	<0.001	0.45	miR-3960	<0.001	-0.45
miR-221-3p	<0.001	0.44	miR-4497	<0.001	-0.40
miR-23a-3p	3.00E-03	0.43	miR-125b-5p	<0.001	-0.31
miR-26a-5p	<0.001	0.36	miR-15b-5p	<0.001	0.29
miR-7-5p	<0.001	0.34	miR-4488	4.44E-03	0.27
miR-100-5p	<0.001	0.27			
miR-3178	<0.001	0.12			

Supplementary Table 2 : miRNAs regulation on steviol treated MKN-45 cells

Reporter name	p-Value	log 2-value	Reporter name	p-Value	log 2-value
miR-1268b	<0.001	19.85	miR-23c	<0.001	-2.05
miR-3607-5p	<0.001	18.55	miR-3197	<0.001	-1.76
miR-151b	<0.001	18.45	miR-5096	<0.001	-1.72
miR-4530	<0.001	2.59	miR-25-3p	<0.001	-1.43
miR-4484	<0.001	2.41	miR-4521	<0.001	-1.33
miR-6125	<0.001	2.29	miR-8485	<0.001	-1.21
miR-7110-5p	<0.001	2.11	miR-23b-3p	<0.001	-1.03
miR-31-5p	<0.001	2.08	miR-92b-3p	<0.001	-0.99
miR-4485-3p	<0.001	1.93	miR-320d	<0.001	-0.96
miR-4497	<0.001	1.89	miR-320b	<0.001	-0.94
miR-4787-5p	<0.001	1.88	miR-23a-3p	<0.001	-0.93
miR-3665	<0.001	1.84	miR-320c	<0.001	-0.86
miR-638	<0.001	1.77	miR-320a	<0.001	-0.84
miR-7704	<0.001	177	miR-191-5p	<0.001	-0.84
miR-4508	<0.001	1.52	miR-24-3p	<0.001	-0.82
miR-1246	<0.001	1.23	miR-4267	<0.001	-0.77
miR-663a	<0.001	1.21	miR-7977	<0.001	-0.75
miR-3960	<0.001	1.14	miR-92a-3p	<0.001	-0.73
miR-6089	<0.001	1.12	miR-181a-5p	<0.001	-0.64
miR-608	<0.001	0.99	miR-30c-5p	<0.001	-0.54
miR-6727-5p	3.68E-03	0.79	miR-30d-5p	<0.001	-0.48
miR-1273g-3p	<0.001	0.69	miR-26a-5p	<0.001	-0.35
miR-7641	<0.001	0.62	miR-30a-5p	<0.001	-034
miR-4734	1.46E-3	0.27	miR-214-3p	<0.001	-0.29
